# Insights into the Interaction of Lysosomal Amino Acid Transporters SLC38A9 and SLC36A1 Involved in mTORC1 Signaling in C2C12 Cells

**DOI:** 10.3390/biom11091314

**Published:** 2021-09-06

**Authors:** Dan Wang, Xuebin Wan, Xiaoli Du, Zhuxia Zhong, Jian Peng, Qi Xiong, Jin Chai, Siwen Jiang

**Affiliations:** 1 Agricultural Ministry Key Laboratory of Swine Breeding and Genetics & Key Laboratory of Agricultural Animal Genetics, Breeding, and Reproduction of Ministry of Education, Huazhong Agricultural University, Wuhan 430070, China; wangdanhzau@163.com (D.W.); wanxuebinhzau@163.com (X.W.); 15207131682@163.com (X.D.); zzx05031319@163.com (Z.Z.); 2 The Cooperative Innovation Center for Sustainable Pig Production, Wuhan 430070, China; 3 Department of Animal Nutrition, Huazhong Agricultural University, Wuhan 430070, China; pengjian@mail.hzau.edu.cn; 4 Hubei Key Laboratory of Animal Embryo Engineering and Molecular Breeding, Institute of Animal Husbandry and Veterinary, Hubei Academy of Agricultural Science, Wuhan 430070, China; phenixxq@163.com

**Keywords:** amino acid transporter, SLC38A9, SLC36A1, lysosome, leucine, mTORC1 signaling pathway

## Abstract

Amino acids are critical for mammalian target of rapamycin complex 1 (mTORC1) activation on the lysosomal surface. Amino acid transporters SLC38A9 and SLC36A1 are the members of the lysosomal amino acid sensing machinery that activates mTORC1. The current study aims to clarify the interaction of SLC38A9 and SLC36A1. Here, we discovered that leucine increased expressions of SLC38A9 and SLC36A1, leading to mTORC1 activation. SLC38A9 interacted with SLC36A1 and they enhanced each other’s expression levels and locations on the lysosomal surface. Additionally, the interacting proteins of SLC38A9 in C2C12 cells were identified to participate in amino acid sensing mechanism, mTORC1 signaling pathway, and protein synthesis, which provided a resource for future investigations of skeletal muscle mass.

## 1. Introduction

Skeletal muscle mass is important to maintain metabolic homeostasis, but protein loss causes a reduction in skeletal muscle mass [1,2]. Muscle protein synthesis is regulated by amino acids, which stimulate mammalian target of rapamycin complex 1 (mTORC1) [3,4,5]. mTORC1 regulates protein translation by phosphorylating ribosomal protein S6 kinase 1 (S6K1) [6], eukaryotic initiation factor 4E-binding protein-1 (4E-BP1) [7], and La-related protein 1 (LARP1) [8]. Leucine (Leu) is a potential signaling molecule that regulates cell growth and metabolism by activating mTORC1. Leu has repeatedly been shown to stimulate protein synthesis in the skeletal muscle [9,10,11]. C2C12 cells are mouse skeletal myoblast cells, which have been used extensively in previous studies on muscle protein synthesis and degradation [12,13].

Lysosome is a key intracellular organelle for mTORC1 activation, protein degradation, and amino acid recycling, and is a site of Ragulator, Rag GTPase, and Rheb localization [14,15]. Amino acid accumulation in the lysosomes can initiate signals to the Ragulator–Rag GTPases complex and regulate the recruitment of mTORC1 to lysosomes, and thus mTORC1 activation [16,17]. Amino acid transporter is the membrane protein that mediates transmembrane transport of amino acids, and interacts functionally with the mTORC1 signaling pathway [18,19]. Several amino acids, including Leu, arginine (Arg), and glutamine (Gln) have been identified as effective activators of mTORC1 [15]. Recent studies have revealed the transport activity of lysosomal amino acid transporter SLC38A9 for Gln [20], Arg [21], and Leu [20,21], and SLC38A9 may itself function as the intracellular amino acid sensor [20,21,22]. It has been reported that SLC38A9 is an upstream regulator of the Ragulator–Rag GTPases complex, which transduces amino acid availability to mTORC1 activity, and the interaction of SLC38A9 and the Ragulator–Rag GTPases complex is modulated by amino acids [15,23]. SLC36A1 is the amino acid transporter on the lysosome, and also named lysosomal amino acid transporter 1 (LYAAT1) [24] and proton-coupled amino acid transporter 1 (PAT1) [25]. SLC36A1 interacts with Rag GTPases and is required for the translocation of mTORC1 to the lysosomal surface [26]. Collectively, SLC38A9 and SLC36A1 play important roles as amino acid transporters on the lysosomal membrane in mTORC1 signaling pathway. However, the interaction of these two lysosomal amino acid transporters remains poorly understood. The aim of the present study is to investigate the interaction of SLC38A9 and SLC36A1, which may constitute a promising lead for potential mechanisms of amino acid sensing and mTORC1 signaling. Further, we aim to identify the interaction proteins of SLC38A9 in C2C12 cells, which may provide a resource for future investigations of amino acid transporters, mTORC1 signaling, protein synthesis, and skeletal muscle mass.

## 2. Materials and Methods

### 2.1. Special Cell Culture Medium Treatment

The C2C12 mouse myoblasts cell line was purchased from the Type Culture Collection of Chinese Academy of Sciences (Shanghai, China). C2C12 cells were cultured in 10% FBS (Gibco, Gaithersburg, MD, USA) in DMEM (Hyclone, South Logan, UT, USA), and they were performed as described previously [27]. After pretreatment without serum for 15 h and without amino acids for 3 h, cells were cultured for 10 min in the special medium (Supplemental Table S1) including the no amino acids group (−AA), the only Arg group (+Arg), the only Leu group (+Leu), and the only Gln group (+Gln). All of the special medium treatments contained the physiological concentration of amino acids found in pig plasma [27,28,29,30].

### 2.2. Immunofluorescence

Coverslips were placed in 6-well plates, and C2C12 myoblasts were seeded into the plates and fixed in 4% paraformaldehyde. Cells were permeabilized with 0.5% Triton X-100 in phosphate-buffered saline (PBS), followed by blocking in PBS containing bovine serum albumin (BSA) and staining with antibodies. The primary antibodies were against SLC38A9 (1:200, ab130398, Abcam, Cambridge, MA, USA), SLC36A1 (1:100, sc-368553, Santa Cruz Biotechnology, Dallas, TX, USA), mTOR (1:100, A2445, Abclonal Technology, Wuhan, China; 1:200, 2983, Cell Signaling Technology, Danvers, MA, USA), and LAMP2 (1:100, sc-20004, Santa Cruz Biotechnology, Dallas, TX, USA). Secondary fluorochrome-conjugated antibodies (Boster Biological Technology, Wuhan, China) were diluted at 1:100. To assess SLC38A9, SLC36A1, and mTOR translocation, the numbers of SLC38A9, SLC36A1, mTOR, and LAMP2-positive spots per cell were calculated using IPP6.0 and Image J software.

### 2.3. Immunoblotting

Protein samples were separated by 10% or 12% SDS-PAGE (SDS, sodium dodecyl sulfate; PAGE, polyacrylamide gel electrophoresis) gels and transferred to a polyvinylidene fluoride (PVDF) membrane. After blocking with 5% fat-free milk in TBST for 2 h, the membranes were incubated with primary antibodies against SLC38A9 (1:1000, ab81687, Abcam, Cambridge, MA, USA), SLC36A1(1:500, sc-368553, Santa Cruz Biotechnology, Dallas, TX, USA), mTOR (1:500, A2445, Abclonal Technology, Wuhan, China), phospho-mTOR (1:500, 2971, Cell Signaling Technology, Danvers, MA, USA), S6K (1:500, sc-230, Santa Cruz Biotechnology, Dallas, TX, USA), phospho-S6K (1:1000, AF3228, Affinity Biosciences, Cincinnati, OH, USA), HA-Tag (1:500, 3724, Cell Signaling Technology, Danvers, MA, USA), and β-Actin (1:1000, BM0627, Boster Biological Technology, Wuhan, China) overnight at 4 °C. The results were visualized using horseradish peroxidase-conjugated secondary antibodies (Santa Cruz Biotechnology, Dallas, TX, USA) and enhanced chemiluminescence.

### 2.4. RNA Extraction, cDNA Synthesis, and Quantitative Real-Time PCR (qRT-PCR)

Total RNA was isolated using an HP Total RNA Kit (Omega, Norcross, GA, USA) in accordance with the manufacturer’s instruction. For mRNA quantification, cDNA was synthesized using a RevertAid First Strand cDNA Synthesis Kit (Thermo Scientific, Waltham, MA, USA) in accordance with the manufacturer’s instruction. qRT-PCR was performed in triplicate using the IQ SYBR green Supermix (BioRad, Hercules, CA, USA) on a CFXTM384 Touch qPCR system (BioRad, Hercules, CA, USA). Relative quantification was performed by the 2^−(ΔΔCt)^ method and normalized by β-Actin. The following primers were used: SLC38A9 (mouse): TGTCATTCAGAGGGTTAGT (sense), TCTTGAGTTTATAGGCAGAG (antisense); SLC36A1 (mouse): GGCAGCATCACCCTCAAC (sense), CACAAAGGTCCACCATCA (antisense); β-Actin (mouse): CTGGCTGGCCGGGACCTGAC (sense), CCGCTCGTTGCCAATAGTGATGAC (antisense); SLC38A9 (pig): CTTTGGGCAGTGGTCGAGTCTTC (sense), GGCACTTGGACAAATCACAGGGT (antisense); SLC36A1 (pig): CGACTTCGGAACGAGGACTAC (sense), GGGTCTGGAACCATGTTGTG (antisense); and β-Actin (pig): CCAGGTCATCACCATCGG (sense), CCGTGTTGGCGTAGAGGT (antisense).

### 2.5. Plasmid Construction, RNA Interference, and Transient Transfection

SLC38A9 and SLC36A1 overexpression constructs were amplified from mouse cDNA and cloned into pcDNA3.1. Small interfering RNA (siRNA) targeting SLC38A9 or SLC36A1 was obtained from GenePharma (Shanghai, China). For mouse, the sequences of SLC38A9 siRNA were 5′-GAUGGGAACAUCUAUACUATT-3′ and 5′-UAGUAUAGAUGUUCCCAUCTT-3′, and the sequences of SLC36A1 siRNA were 5′-GUGUGCUCAUCACUUAUGUTT-3′ and 5′-ACAUAAGUGAUGAGCACACTT-3′. For pig, the sequences of SLC38A9 siRNA were 5′-GCAGUGACAUCCUGUCCUUTT-3′ and 5′-AAGGACAGGAUGUCACUGCTT-3′, and the sequences of SLC36A1 siRNA were 5′-CCGAGAUCAUCAUUCCUUUTT-3′ and 5′-AAAGGAAUGAUGAUCUCGGTT-3′. For the SLC38A9 and SLC36A1 overexpression experiments, C2C12 cells were transfected with a pcDNA3.1 expression plasmid expressing SLC38A9 or SLC36A1, or an empty pcDNA3.1 plasmid using Lipofectamine 2000 (Invitrogen, Carlsbad, CA, USA), and they were harvested 48 h later. In the SLC38A9 and SLC36A1 knockdown studies, C2C12, 3T3-L1, BHK-21, PK-15, and ST cells were either transfected with control siRNA (NC) or SLC38A9 and SLC36A1 siRNA using Lipofectamine 2000 (Invitrogen, Carlsbad, CA, USA), and they were harvested 48 h later. The 3T3-L1 and BHK-21 cell lines were purchased from the Type Culture Collection of Chinese Academy of Sciences (Shanghai, China). PK-15 and ST cell lines were purchased from the China Center for Type Culture Collection (Wuhan, China).

### 2.6. Co-Immunoprecipitation (Co-IP)

C2C12 cells were washed with PBS and lysed using lysis buffer (P0013, Beyotime Biotechnology, Shanghai, China) supplemented with protease inhibitor and protein phosphorylase inhibitor on ice for 15 min. The lysates were centrifuged for 10 min at 10,000 rpm to pellet the cell debris and precleared by addition of protein A/G plus agarose beads and incubation with rotation for 2 h at 4 °C. Agarose beads were removed by centrifugation, and the supernatant was incubated by the antibody against SLC36A1 (1:20, sc-161150, Santa Cruz Biotechnology, Dallas, TX, USA) or HA-Tag (1:25, AE008, Abclonal Technology, Wuhan, China) with rotation overnight at 4 °C and followed by the addition of agarose beads with rotation for 6 h at 4 °C. After washing three times with lysis buffer, proteins were eluted by loading buffer and boiled for 10 min.

### 2.7. MS-Based Proteomics

The SLC38A9 overexpression construct was amplified from mouse cDNA and then cloned into pCMV-HA. Expression of HA-tagged SLC38A9 or pCMV-HA in C2C12 cells was separately induced for 48 h. Cells were washed with PBS and lysed in lysis buffer (P0013, Beyotime Biotechnology, Shanghai, China) supplemented with protease inhibitor and protein phosphorylase inhibitor. Cell debris was removed from the lysates by centrifugation. For immunoprecipitation, the lysates were precleared by addition of protein A/G plus agarose beads and incubation with rotation for 2 h at 4 °C. Agarose beads were removed by centrifugation, and the supernatant was incubated by the antibody against HA-Tag (1:50, 3724, Cell Signaling Technology, Danvers, MA, USA) with rotation overnight at 4 °C and followed by the addition of agarose beads with rotation for 6 h at 4 °C. Proteins were eluted by loading buffer, boiled for 10 min, and separated by 10% SDS-PAGE gel and stained with Coomassie brilliant blue. Differential bands were cut for analysis by liquid chromatography/mass spectrometry (LC-MS/MS).

### 2.8. Functional Annotation and Enrichment Analysis

Functional annotation and enrichment analysis were applied to the proteins using the Gene Ontology (GO) database, the Kyoto Encyclopedia of Genes and Genomes (KEGG) database, and protein–protein interaction (PPI) data. The GO database included biological processes, cellular components, and molecular functions. The KEGG database contained the integrated information about the genome, chemistry, and systemic function. PPI analysis was performed using the STRING database and Cytoscape 3.6.1 software.

### 2.9. Statistical Analysis

All statistical analyses were conducted using GraphPad Prism 6 and Excel 2013. Results were presented as mean ± SEM. Significance of differences was calculated with a *t*-test and *p*-values of < 0.05 were considered significant.

## 3. Results

### 3.1. Leucine Upregulates the Activity of mTORC1 and the Expression of SLC38A9 and SLC36A1

**Figure 1:**
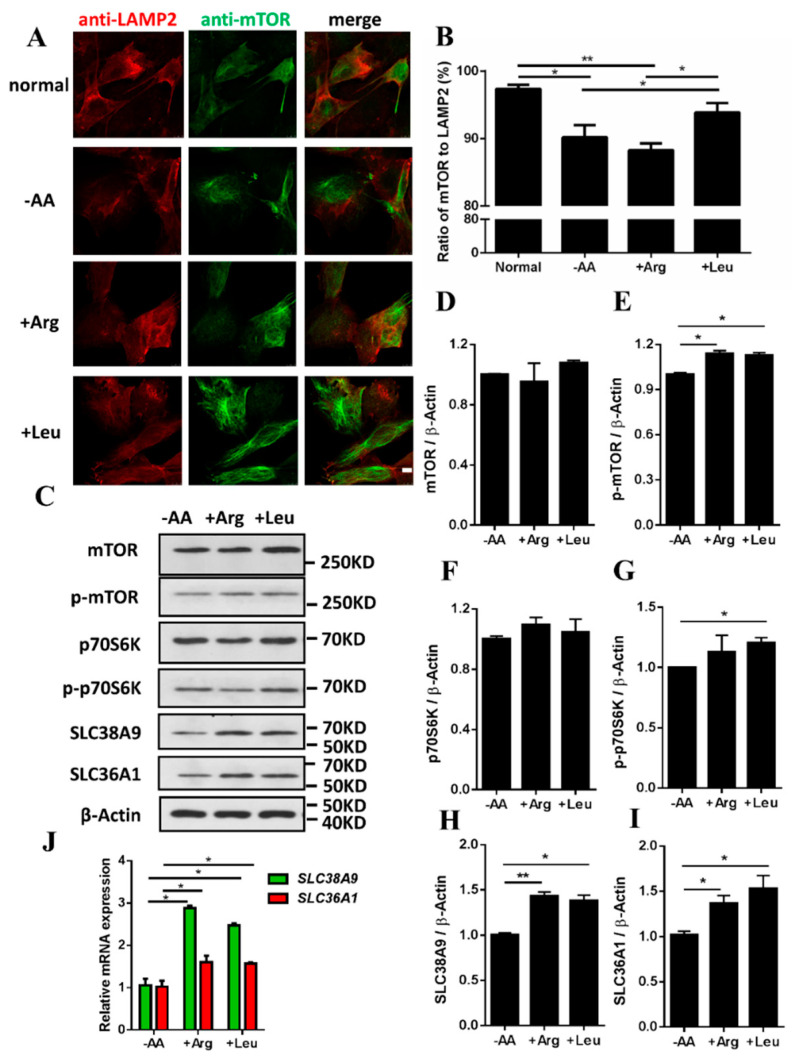
Effects of Arg and Leu on the location of mTOR on LAMP2-positive lysosomal clusters, activation of mTORC1 and expression of SLC38A9 and SLC36A1 in C2C12 cells. (**A**) C2C12 cells were pretreated without serum for 15 h and without amino acids for 3 h then cultured for 10 min in a special medium (Supplemental Table S1) including the no amino acids group (−AA), the only Arg group (+Arg), and the only Leu group (+Leu). Cells were stained with anti-mTOR (A2445, Abclonal Technology, Wuhan, China) or anti-LAMP2. Scale bar: 10 µM. (**B**) Ratio of mTOR to LAMP2 (%). To assess mTOR translocation, the numbers of mTOR and LAMP2-positive spots per cell were calculated using IPP6.0 and Image J software. (**C**) Immunoblotting analysis of protein samples from (**A**) with anti-mTOR, anti-phospho-mTOR, anti-S6K, anti-phospho-S6K, anti-SLC38A9, anti-SLC36A1, or anti-β-Actin antibody. (**D**) Densitometric analysis of the immunodetection of mTOR relative to β-Actin loading control. (**E**) Densitometric analysis of the immunodetection of phospho-mTOR relative to β-Actin loading control. (**F**) Densitometric analysis of the immunodetection of p70S6K relative to β-Actin loading control. (**G**) Densitometric analysis of the immunodetection of phospho-p70S6K relative to β-Actin loading control. (**H**) Densitometric analysis of the immunodetection of SLC38A9 relative to β-Actin loading control. (**I**) Densitometric analysis of the immunodetection of SLC36A1 relative to β-Actin loading control. (**J**) The mRNA levels of SLC38A9 and SLC36A1 were analyzed by qRT-PCR. Values are the mean ± SEM; *n* = 3; * *p* < 0.05; ** *p* < 0.01.

Endogenous mTOR localized to LAMP2-positive lysosomal clusters under a normal state. Withdrawal of amino acids inhibited lysosomal localization of mTOR, but mTOR was translocated to LAMP2-positive lysosomal clusters upon the addition of Leu after amino acid starvation (Figure 1A,B and Supplemental Figure S1). mTOR and S6K phosphorylation increased upon the addition of Leu after amino acid starvation (Figure 1C–G). Arg increased mTOR phosphorylation but had no effect on the recruitment of mTOR to lysosome (Figure 1A–E). This suggests that mTORC1 activity and location of mTOR on LAMP2-positive lysosomal clusters were enhanced by the addition of Leu after amino acid starvation. Since SLC38A9 and SLC36A1 are lysosomal amino acid transporters and regulate mTORC1 activity in an amino acid-dependent manner, we evaluated whether Arg and Leu affected the expression of SLC38A9 and SLC36A1. The mRNA and protein levels of SLC38A9 and SLC36A1 increased upon the addition of Arg or Leu after amino acid starvation (Figure 1C,H–J). Gln increased mTOR and S6K phosphorylation, but Gln had no effect on the mRNA and protein levels of SLC38A9 and SLC36A1 (Supplemental Figure S2). It indicated that Gln might regulate mTORC1 activity not through SLC38A9 or SLC36A1. These results indicated that amino acid transporters SLC38A9 and SLC36A1 may have essential roles in the Leu induced mTORC1 signaling pathway.

### 3.2. SLC38A9 and SLC36A1 Increase mTOR Phosphorylation

**Figure 2:**
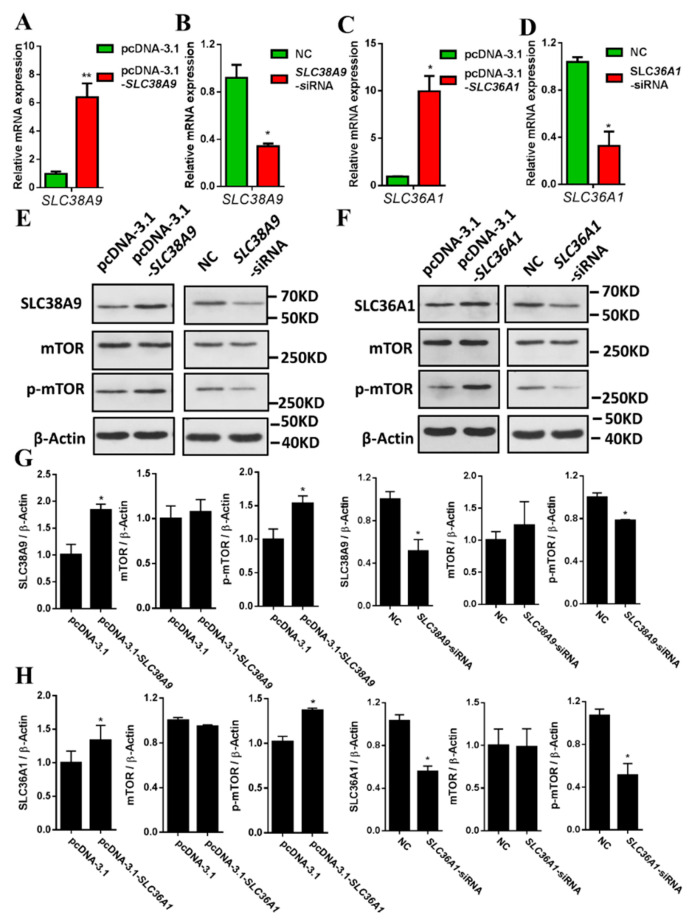
The effects of SLC38A9 or SLC36A1 overexpression and inhibition on mTOR phosphorylation in C2C12 cells. (**A**) The mRNA expression of SLC38A9 after overexpression of SLC38A9. (**B**) The mRNA expression of SLC38A9 after inhibition of SLC38A9. (**C**) The mRNA expression of SLC36A1 after overexpression of SLC36A1. (**D**) The mRNA expression of SLC36A1 after inhibition of SLC36A1. (**E**) C2C12 cells were transfected with pcDNA3.1-SLC38A9 construct or siRNA to overexpress or knockdown SLC38A9. Immunoblotting analysis of protein samples with anti-SLC38A9, anti-mTOR, anti-phospho-mTOR, or anti-β-Actin antibody. (**F**) C2C12 cells were transfected with pcDNA3.1-SLC36A1 construct or siRNA to overexpress or knockdown SLC36A1. Immunoblotting analysis of protein samples with anti-SLC36A1, anti-mTOR, anti-phospho-mTOR, or anti-β-Actin antibody. (**G**) Densitometric analysis of the immunodetection of SLC38A9, mTOR or phospho-mTOR relative to β-Actin loading control from (**E**). (**H**) Densitometric analysis of the immunodetection of SLC36A1, mTOR or phospho-mTOR relative to β-Actin loading control from (**F**). Values are the mean ± SEM; *n* = 3; * *p* < 0.05; ** *p* < 0.01.

Knockdown of SLC38A9 or SLC36A1 reduces phosphorylation of S6K1 and 4E-BP1 [21,23,31,32,33]. SLC38A9 and SLC36A1 may have essential roles in mTORC1 signaling pathway. mTORC1 contains mTOR phosphorylated at Serine 2448 (Ser2448) which is a target of S6K1 [34,35,36]. Ser2448 phosphorylation is measured to assess mTOR kinase activation or as a measurement of induction of the mTOR pathway in muscle tissue and is implicated in mTORC1 activity [34,37,38,39]. The effects of SLC38A9 and SLC36A1 on mTOR phosphorylation at Ser2448 in C2C12 cells remains unclear. Mouse SLC38A9 cDNA was cloned into pcDNA3.1, the overexpression of SLC38A9 in C2C12 cells increased the mRNA and protein levels of SLC38A9 (Figure 2A,E,G). After transfection of the C2C12 cells with individual siRNAs targeted to knockdown SLC38A9, the inhibition of SLC38A9 suppressed the mRNA and protein levels of SLC38A9 (Figure 2B,E,G). The results of overexpression and inhibition of SLC36A1 are shown in Figure 2. The overexpression of SLC38A9 or SLC36A1 in C2C12 cells promoted the phosphorylation of mTOR at Ser2448, while the inhibition of SLC38A9 or SLC36A1 had the opposite effect (Figure 2E–H). This result suggested that SLC38A9 and SLC36A1 upregulate mTOR phosphorylation at Ser2448.

### 3.3. Interaction of Amino Acid Transporters SLC38A9 and SLC36A1

**Figure 3:**
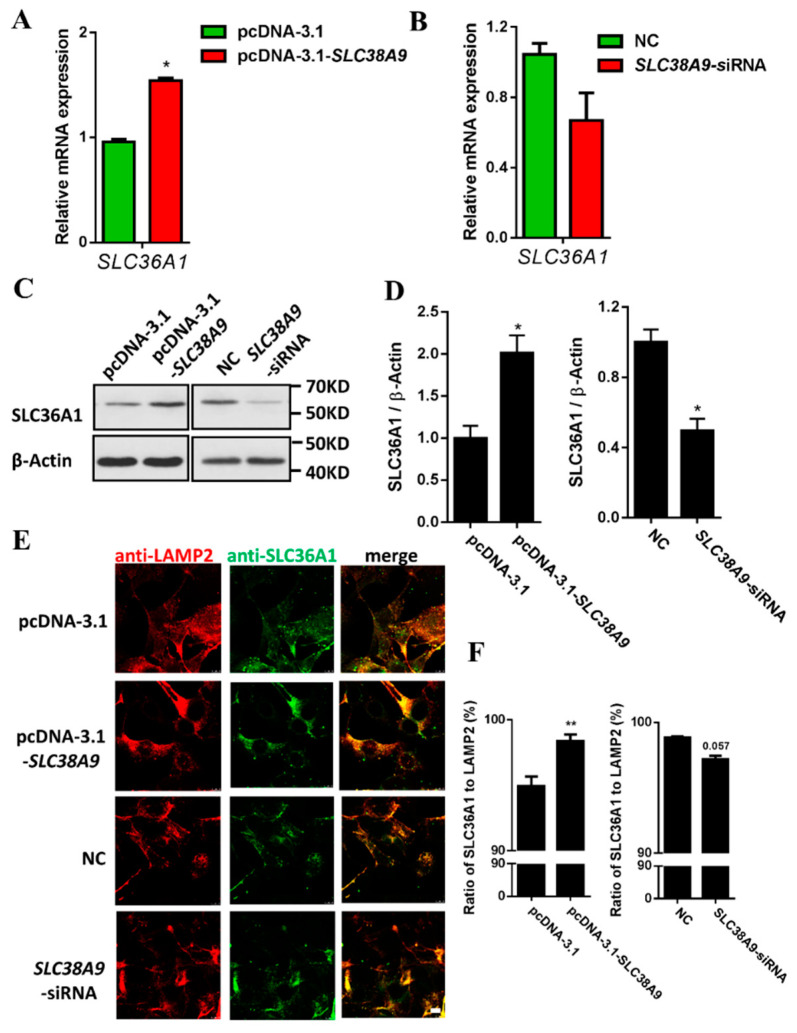
SLC38A9 increases SLC36A1 expression and promotes the translocation of SLC36A1 to LAMP2-positive lysosomal clusters. (**A**) C2C12 cells were transfected with pcDNA3.1-SLC38A9 construct to overexpress SLC38A9. The mRNA level of SLC36A1 was analyzed by qRT-PCR. (**B**) C2C12 cells were transfected with siRNA to knockdown SLC38A9. The mRNA level of SLC36A1 was analyzed by qRT-PCR. (**C**) Immunoblotting analysis of protein samples from (**A**,**B**) with anti-SLC36A1 or anti-β-Actin antibody. (**D**) Densitometric analysis of the immunodetection of SLC36A1 relative to β-Actin loading control. (**E**) C2C12 cells were transfected with pcDNA3.1-SLC38A9 construct or siRNA to overexpress or knockdown SLC38A9 and stained with anti-SLC36A1 or anti-LAMP2. Scale bar: 10µM. (**F**) Ratio of SLC36A1 to LAMP2 (%). To assess SLC36A1 translocation, the numbers of SLC36A1 and LAMP2-positive spots per cell were calculated using IPP6.0 and Image J software. Values are the mean ± SEM; *n* = 3; * *p* < 0.05; ** *p* < 0.01.

SLC38A9 and SLC36A1 play important roles in the mTORC1 signaling pathway, and they are both amino acid transporters on the lysosomal membrane. This study aimed to investigate the relationship of SLC38A9 and SLC36A1. The overexpression of SLC38A9 in C2C12 myoblasts promoted the mRNA and protein levels of SLC36A1 (Figure 3A,C,D). After transfection of the C2C12 myoblasts with individual siRNAs targeted to knockdown SLC38A9, the inhibition of SLC38A9 suppressed the protein level of SLC36A1 (Figure 3C,D). To confirm the effect of SLC38A9 on SLC36A1 expression, individual siRNAs were transfected, respectively, to knockdown SLC38A9 in different cell lines (3T3-L1, BHK-21, PK-15 and ST). The mRNA level of SLC36A1 decreased in 3T3-L1 and PK-15 cells (Supplemental Figure S3A). The inhibition of SLC38A9 suppressed the protein level of SLC36A1 (Supplemental Figure S3B,D). Endogenous SLC38A9 and SLC36A1 were localized to LAMP2-positive lysosomal clusters and their locations on LAMP2-positive lysosomal clusters were increased by overexpression and decreased by inhibition (Supplemental Figure S4). This revealed that the staining of antibody was specific for SLC38A9 or SLC36A1. The overexpression of SLC38A9 promoted the translocation of SLC36A1 to LAMP2-positive lysosomal clusters, while the ratio of SLC36A1 to LAMP2 showed a downward trend by the inhibition of SLC38A9 (Figure 3E,F). These results indicate that the expression of SLC38A9 affects SLC36A1 expression and location.

**Figure 4:**
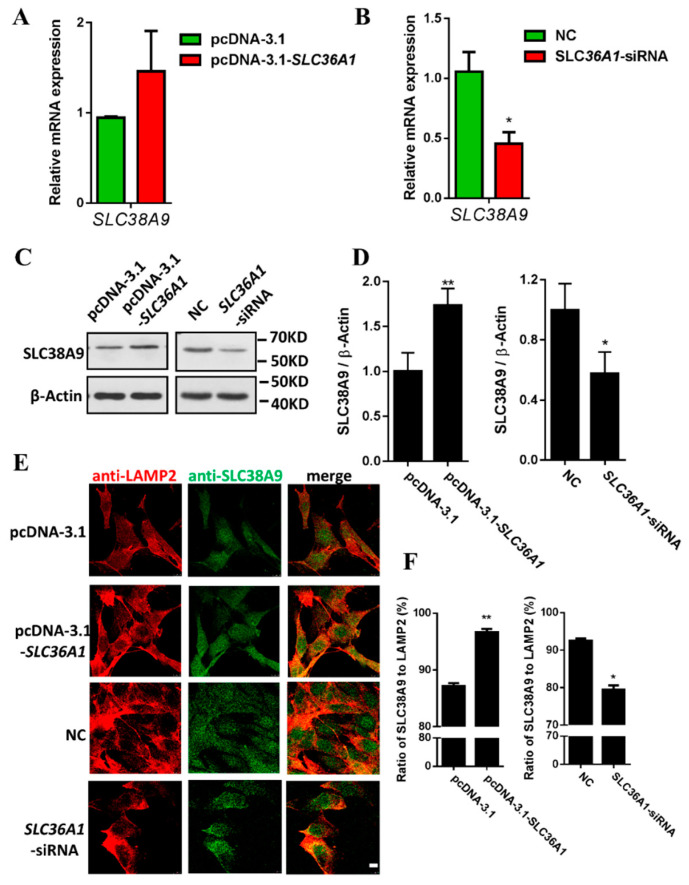
SLC36A1 increases SLC38A9 expression and promotes the translocation of SLC38A9 to LAMP2-positive lysosomal clusters. (**A**) C2C12 cells were transfected with pcDNA3.1-SLC36A1 construct to overexpress SLC36A1. The mRNA level of SLC38A9 was analyzed by qRT-PCR. (**B**) C2C12 cells were transfected with siRNA to knockdown SLC36A1. The mRNA level of SLC38A9 was analyzed by qRT-PCR. (**C**) Immunoblotting analysis of protein samples from (**A**,**B**) with anti-SLC38A9 or anti-β-Actin antibody. (**D**) Densitometric analysis of the immunodetection of SLC38A9 relative to β-Actin loading control. (**E**) C2C12 cells were transfected with pcDNA3.1-SLC36A1 construct or siRNA to overexpress or knockdown SLC36A1 and stained with anti-SLC38A9 or anti-LAMP2. Scale bar: 10µM. (**F**) Ratio of SLC38A9 to LAMP2 (%). To assess SLC38A9 translocation, the numbers of SLC38A9 and LAMP2-positive spots per cell were calculated using IPP6.0 and Image J software. Values are the mean ± SEM; *n* = 3; * *p* < 0.05; ** *p* < 0.01.

Then we tested whether the expression of SLC36A1 affected SLC38A9 expression and location. The inhibition of SLC36A1 suppressed the mRNA and protein levels of SLC38A9 (Figure 4B–D). The overexpression of SLC36A1 promoted the protein level of SLC38A9 (Figure 4C,D). To further confirm the effect of SLC36A1 on SLC38A9 expression, individual siRNAs were transfected to knockdown SLC36A1 in different cell lines (3T3-L1, BHK-21, PK-15 and ST). The mRNA level of SLC38A9 decreased in 3T3-L1, BHK-21, and PK-15 cells (Supplemental Figure S3E). The inhibition of SLC36A1 suppressed the protein level of SLC38A9 (Supplemental Figure S3F,H). Translocation of SLC38A9 to LAMP2-positive lysosomal clusters was increased by the overexpression of SLC36A1 and decreased by the inhibition of SLC36A1 (Figure 4E,F). These results revealed SLC38A9 and SLC36A1 influenced each other in expression and location.

**Figure 5:**
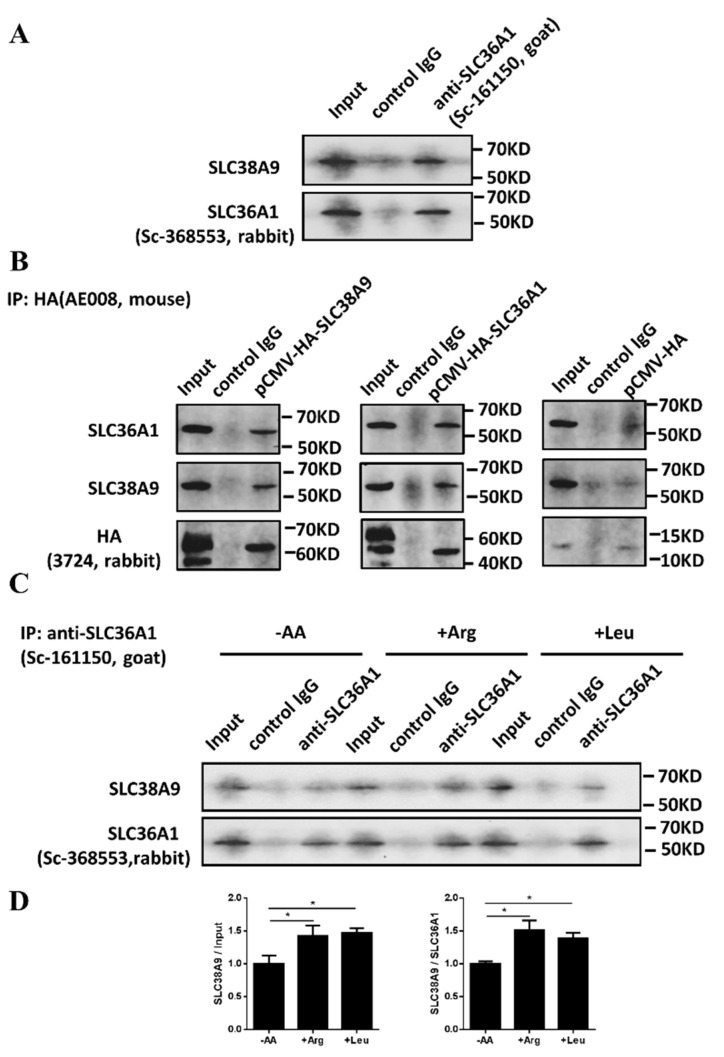
SLC38A9 and SLC36A1 interact. (**A**) SLC38A9 was associated with SLC36A1 at the endogenous level as detected by Co-IP: anti-SLC36A1 (sc-161150, Santa Cruz) was used for pull-down, and anti-SLC38A9 (ab81687, Abcam) and anti-SLC36A1 (sc-368553, Santa Cruz) were used for detection. (**B**) Interaction of SLC38A9 and SLC36A1: C2C12 cells were transfected with SLC38A9 or SLC36A1 in the pCMV-HA vector, and the lysates were prepared and subjected to HA immunoprecipitation followed by immunoblotting for the indicated proteins. (**C**) C2C12 cells were pretreated without serum for 15 h, and without amino acids for 3 h, then cultured for 10 min in a special medium (Supplemental Table S1) including the no amino acids group (−AA), the only Arg group (+Arg), the only Leu group (+Leu). The interaction of SLC38A9 and SLC36A1 was detected by Co-IP as (**A**). (**D**) Densitometric analysis of the immunodetection of SLC38A9 relative to input control or SLC36A1. Values are the mean ± SEM; *n* = 3; * *p* < 0.05.

SLC38A9 and SLC36A1 influenced each other in expression and location, and they are both on the lysosomal membrane. We evaluated whether SLC38A9 and SLC36A1 interact. It was validated by Co-IP and immunoblotting. Co-IP with the anti-SLC36A1 antibody confirmed the association of SLC38A9 with SLC36A1 at the endogenous level (Figure 5A). Expression of HA-tagged SLC38A9 or HA-tagged SLC36A1 was separately induced in C2C12 cells for 48 h, and Co-IP with the anti-HA-Tag antibody confirmed the association between SLC38A9 and SLC36A1. pCMV-HA was used as the negative control (Figure 5B). Since SLC38A9 was interacted with SLC36A1 at the endogenous level (Figure 5A), the association between the two amino acid transporters in amino acid starvation and presence of Arg or Leu was measured. The interaction of SLC38A9 and SLC36A1 was enhanced by the addition of Arg or Leu after amino acid starvation (Figure 5C,D). Collectively, these findings suggest that SLC38A9 and SLC36A1 interacted, and the interaction was affected by Arg and Leu.

### 3.4. Identification of Interacting Proteins of SLC38A9

**Figure 6:**
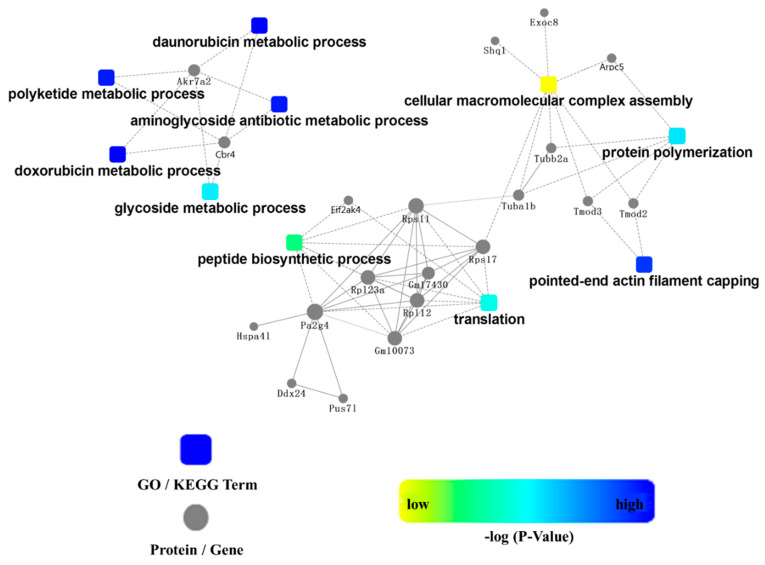
PPI network. Proteins are represented by dots and signaling pathways are indicated by rounded rectangles.

To perform interaction proteomics within SLC38A9, C2C12 cells were transfected with HA-tagged SLC38A9 or empty plasmid pCMV-HA for 48 h. Lysates derived from cells were subjected to parallel HA-immunoprecipitations, isolated by SDS–PAGE (Supplemental Figure S5), and followed by peptide elution, trypsin digestion, and analysis by LC-MS/MS. We identified 62 differentially expressed proteins (Supplemental Table S2). They were classified by functional category using GO annotations, and GO analysis was performed to determine their biological processes, cell components, and molecular functions. The GO terms in the biological process included pointed-end actin filament capping, protein polymerization, translation, and peptide biosynthetic process, as well as cellular macromolecular complex assembly, myofibril assembly, striated muscle cell development, muscle cell development, and cellular protein complex assembly. The GO terms in the cell component included ribosome, cytosolic, ribonucleoprotein complex, TOR complex, and myofibril. The GO terms in the molecular function were involved in the structural constituent of ribosome and cytoskeleton, tropomyosin binding, rRNA binding, RNA binding, RAGE receptor binding, and oxidoreductase activity (Supplemental Figure S6A). The KEGG pathway analysis showed that the most enriched pathways were associated with amino acid sensing and protein synthesis, such as the mTOR signaling pathway, ribosome, protein processing in endoplasmic reticulum, glutathione metabolism, and the AMPK signaling pathway (Supplemental Figure S6B). Figure 6 shows the network analysis of proteins and signaling pathways. Peptide biosynthetic process and translation related proteins included EIF2AK4, RPS11, RP123A, GM17430, RPS17, HSPA41, PA2G4, RP112, GM10073, DDX24, and PUS71. The proteins related to cellular macromolecular complex assembly, protein polymerization, and pointed-end actin filament capping were EXOC8, SHQ1, ARPC5, TUBB2A, TUBA1B, TMOD3, and TMOD2. These results provide a resource for future investigations of amino acid transporters, mTORC1 signaling, and protein synthesis.

## 4. Discussion

Our previous study associated a low protein diet with high expression of amino acid transporters and the inhibition of the mTORC1 activity, which restricted protein synthesis and *longissimus dorsi* growth in pigs. Furthermore, in vitro experimental results confirmed that the mRNA levels of amino acid transporters increased and the phosphorylation of mTOR and S6K1 decreased when the concentration of amino acids in C2C12 myoblasts was reduced [27]. In this study, we used C2C12 cells to study the effect of amino acid transporters on the mTORC1 signaling pathway, which might play a role in the regulation of protein synthesis and thus skeletal muscle mass.

Lysosomes are recognized as key intracellular organelles in mTORC1 activation by amino acids [17], as they regulate the mTORC1 signaling pathway by providing a surface for the formation of the Ragulator–Rag GTPases complex [16,40]. Leu functions not only as a substrate for protein synthesis but also as a signaling molecule for the initiation of protein synthesis and regulation of the translocation of mTOR to the surface of lysosome where it is directly activated [41,42]. Arg is required for mTORC1 activation [43]. mTORC1 signaling is activated by replenishment of individual amino acids (Arg, Leu, or Gln) for 10 min after amino acid starvation [20,21,23]. In this study, mTORC1 activation was enhanced and mTOR was significantly retained on the lysosomal membrane upon the addition of Leu after amino acid starvation (Figure 1 and Supplemental Figure S1). SLC38A9 functions upstream of the Ragulator–Rag GTPases complex and is an excellent candidate for the Arg sensor in the mTORC1 signaling pathway [21,44]. SLC36A1 localizes to the lysosome and is involved in amino acid-dependent processes that localize and activate mTORC1 [24,26,45]. In this study, the expression of SLC38A9 and SLC36A1 was increased with the addition of Arg or Leu after amino acid starvation (Figure 1). In our previous study, addition of Leu after amino acid starvation increased the mRNA expression of SLC38A9 in porcine skeletal muscle cells [29]. SLC38A9 and SLC36A1 may regulate mTORC1 activity in an amino acid-dependent manner [15]. Gln increased mTOR and S6K1 phosphorylation, but it had no effect on the mRNA and protein levels of SLC38A9 and SLC36A1 (Supplemental Figure S2). It has been reported that Gln stimulates mTORC1 by Rag GTPase-independent mechanism [46]. Gln may regulate mTORC1 activity not through SLC38A9 or SLC36A1. SLC38A9 and SLC36A1 are critical for mTORC1 activation [16,20,21,26,47], SLC36A1 is required for mTOR recruitment to the lysosomal surface [26], and SLC38A9 is required to release the inactivated mTOR from the lysosomal membranes [23]. In this study, SLC38A9 and SLC36A1 promoted the phosphorylation of mTOR at Ser2448 (Figure 2), which is implicated in mTORC1 activity in muscle tissue [36,39]. Knockdown of SLC38A9 or SLC36A1 is reported to reduce mTORC1 signaling targets, namely phosphorylated S6K1 and 4E-BP1 [21,23,31,32,33,48]. These findings suggest that the activation of mTORC1 may be upregulated by Leu via SLC38A9 and SLC36A1.

SLC38A9 and SLC36A1 function as intracellular amino acid sensors to regulate the mTORC1 signaling pathway [23,24,26,45], and they are both amino acid transporters on the lysosomal membrane. We are interested in the relationship of these two lysosomal amino acid transporters. In this study, SLC38A9 and SLC36A1 were interacted and influenced each other’s expression and the location on LAMP2-positive lysosomal clusters (Figure 3, Figure 4 and Figure 5). The relationship between the interaction and mTORC1 signaling and the effect of SLC36A1 expression on SLC38A9 function in mTORC1 signaling and vice versa remain unclear. The additional relationship between two transporters still needs to be confirmed in further studies. mTORC1 is activated on lysosomal membranes by the Ragulator–Rag GTPases machinery [41]. SLC38A9 is a component of the Ragulator–Rag GTPases complex, and SLC36A1 physically interacts with Rag GTPases [21,23,26]. Rag GTPases may be involved in the interaction of SLC38A9 and SLC36A1. The interaction was enhanced by the addition of Arg or Leu after amino acid starvation in this study. Leu stimulates mTORC1 by Rag GTPase-dependent mechanism [46], the interaction of SLC38A9 and SLC36A1 may take part in this process. Arg induces the conformational change of SLC38A9 that promotes its interaction with Ragulator–Rag GTPases. SLC38A9 has a high affinity transport for Leu in an Arg regulated manner, and it is inhibited by the elimination of the capacity of Arg-induced conformational change of SLC38A9 [49,50]. The interaction of SLC38A9 and SLC36A1 may be regulated by Arg-induced conformational change of SLC38A9, but it still needs to be investigated. We speculate that SLC38A9 and SLC36A1 are novel components of the Ragulator–Rag GTPases complex and participate in amino acid sensing and mTORC1 signaling. The N-terminal cytoplasmic tail of SLC38A9 (not transmembrane region) is required to bind the Ragulator–Rag GTPases complex and participate in mTORC1 signaling [20,21]. However, the region of SLC36A1 involved in the binding is unclear. Further studies should focus on the region of SLC38A9 and SLC36A1 involved in the binding and the effect of the region on mTORC1 signaling. It may bring additional insights to the interaction of SLC38A9 and SLC36A1.

SLC38A9 is a physical and functional component of the amino acid sensing machinery that controls mTORC1 activity, which might play a role in the regulation of protein synthesis [20,21]. Identification of interaction proteins of SLC38A9 in C2C12 cells may provide a resource for future investigations of amino acid transporters, mTORC1 signaling, protein synthesis, and skeletal muscle mass. In this study, novel proteins associated with SLC38A9 were identified by interaction proteomics. Of the 62 proteins (Supplemental Table S2), those of interest include SLC26A11, PA2G4, RPL12, RPL23A, RPS11, RPS17, TMOD2, TMOD3, AKT1S1, KAT8, and RPS6KC1. SLC26A11 mediates the electrogenic transport of chloride and localizes with H^+^-ATPase [51,52]. H^+^-ATPase is involved in lysosomal biogenesis, is an essential component of the amino acid sensing mechanism, and is necessary for mTORC1 activation by amino acids [17]. EBP1, a member of the PA2G4 family [53], is involved in the regulation of cell growth, differentiation, and apoptosis [54]. Ribosomal proteins RPL12, RPL23A, RPS11, and RPS17 are involved in the regulation of protein synthesis [54,55,56,57,58]. TMOD2 and TMOD3 influence muscle contraction and vesicle-membrane fusion [59,60,61]. AKT1S1 inhibits Rheb-induced activation of the mTORC1 signaling pathway [62]. Knockdown of KAT8 appears to induce autophagy consistent with the inhibition of mTORC1 signaling [63,64]. RPS6KC1 plays an important role in regulating protein degradation, recycling, and secretion [65,66]. Unfortunately, SLC36A1 was not found in the differential proteins obtained by LC-MS/MS. In the previous proteomic analysis of SLC38A9, SLC36A1 was not identified as a specific interactor of SLC38A9 [20]. However, the interaction of SLC36A1 with SLC38A9 was verified by immunoblotting (Figure 5). Based on the GO analysis, the cell components included ribosomes, ribonucleoprotein complexes, and the TOR complex (Supplemental Figure S6A). The KEGG pathway analysis showed that most enriched pathways were associated with amino acid sensing and protein synthesis such as mTOR signaling pathway, ribosomes, protein processing in endoplasmic reticulum, and the AMPK signaling pathway (Supplemental Figure S6B). In the translation system, amino acids are joined into proteins, and this system includes the ribosome and its related factors required for polymerization [67,68]. mTOR consists of two protein complexes with different physiologic functions: mTORC1 and mTORC2. mTORC1 regulates cell metabolism by promoting protein synthesis and inhibiting autophagy [69,70,71]. Amino acids regulate muscle protein synthesis via cellular signaling pathways involving mTORC1 and AMPK [72]. AMPK can regulate protein synthesis, apoptosis, and autophagy [48,73,74,75,76]. These results demonstrate that the interaction proteins of SLC38A9 may take part in the amino acid sensing mechanism, mTORC1 signaling pathway, and protein synthesis and degradation.

## 5. Conclusions

Leucine increases expressions of SLC38A9 and SLC36A1, which may lead to the activation of mTORC1. SLC38A9 and SLC36A1 interact and influence each other’s expression levels and locations on the lysosomal surface. One of the next important steps will be determining the region of SLC38A9 and SLC36A1 involved in the binding and the relationship between the interaction and mTORC1 signaling. Interaction proteins of SLC38A9 in C2C12 cells take part in the regulation of mTORC1 signaling pathway and protein synthesis, which provides a resource for future investigations of skeletal muscle mass.

## Data Availability

The data presented in this study are available in the article and Supplementary Material.

## Supplementary Materials

The following are available online at https://www.mdpi.com/article/10.3390/biom11091314/s1, Figure S1: Effects of Arg and Leu on the location of mTOR on LAMP2-positive lysosomal clusters in C2C12 cells. Figure S2: Glutamine (Gln) increases mTOR and S6K1 phosphorylation and has no effect on the mRNA and protein levels of SLC38A9 and SLC36A1, Figure S3: Inhibition of SLC38A9 or SLC36A1 suppressed the mRNA and protein levels of SLC36A1 and SLC38A9 in different cell lines, Figure S4: Effects of the overexpression or inhibition of SLC38A9 and SLC36A1 on the location of SLC38A9 or SLC36A1 on LAMP2-positive lysosomal clusters. Figure S5: Isolation of HA-immunoprecipitation proteins by SDS-PAGE, Figure S6: Significantly enriched GO terms and enriched KEGG pathways, Table S1: Amino acids composition in special cell culture medium, Table S2: Proteomic data of differentially expressed proteins.
