# Supplementary material for: Insights into the Interaction of Lysosomal Amino Acid Transporters SLC38A9 and SLC36A1 Involved in mTORC1 Signaling in C2C12 Cells
Source: Biomolecules. 2021 Sep 6;11(9):1314. doi: 10.3390/biom11091314 (PMC8467208; doi:10.3390/biom11091314)
Supplement: Supplementary file 1 [file biomolecules-11-01314-s001.zip › Supplemental Figures.pptx]

## Slide 1
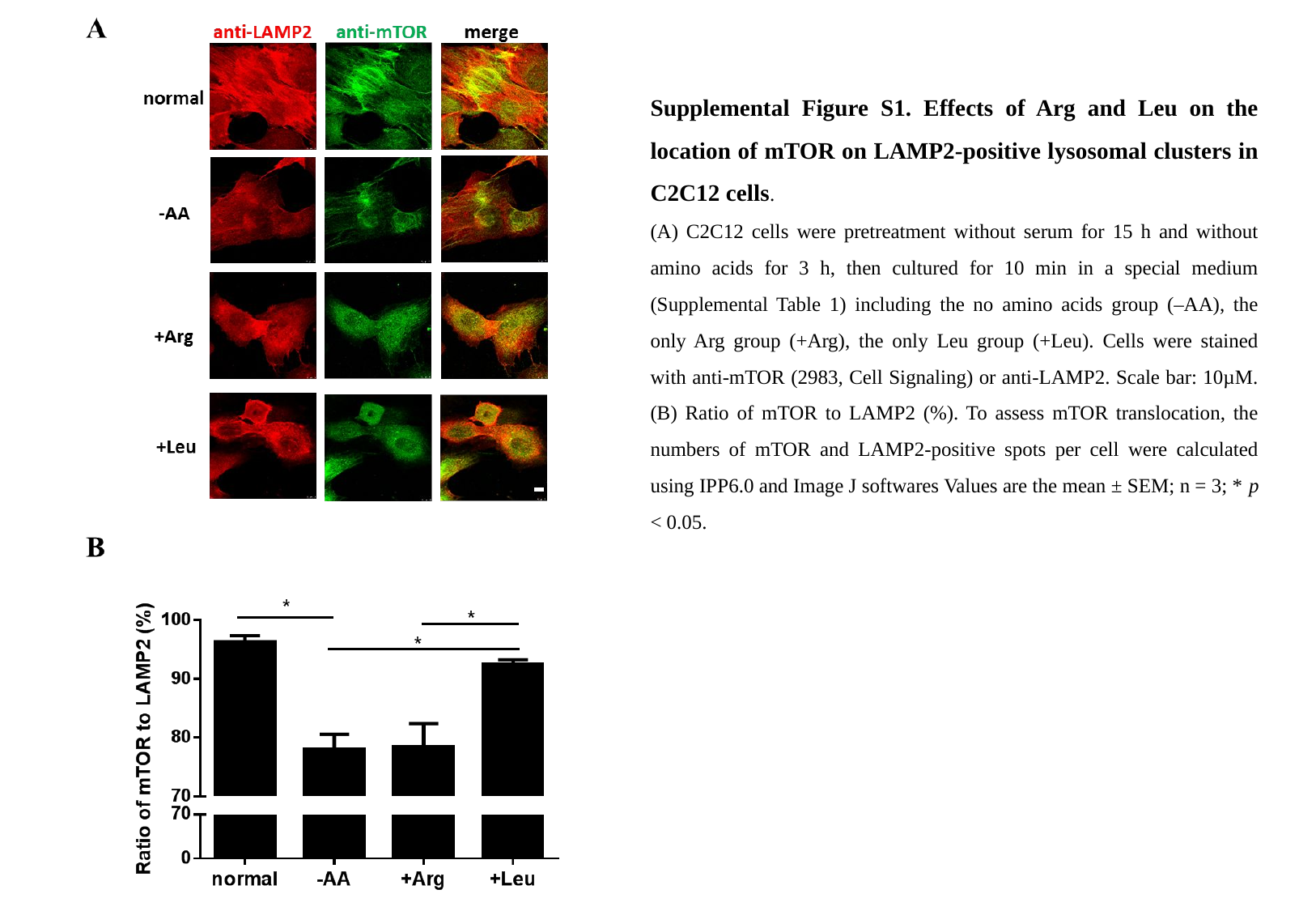

Supplemental Figure S1. Effects of Arg and Leu on the location of mTOR on LAMP2-positive lysosomal clusters in C2C12 cells.
(A) C2C12 cells were pretreatment without serum for 15 h and without amino acids for 3 h, then cultured for 10 min in a special medium (Supplemental Table 1) including the no amino acids group (–AA), the only Arg group (+Arg), the only Leu group (+Leu). Cells were stained with anti-mTOR (2983, Cell Signaling) or anti-LAMP2. Scale bar: 10µM. (B) Ratio of mTOR to LAMP2 (%). To assess mTOR translocation, the numbers of mTOR and LAMP2-positive spots per cell were calculated using IPP6.0 and Image J softwares Values are the mean ± SEM; n = 3; * p < 0.05.

## Slide 2
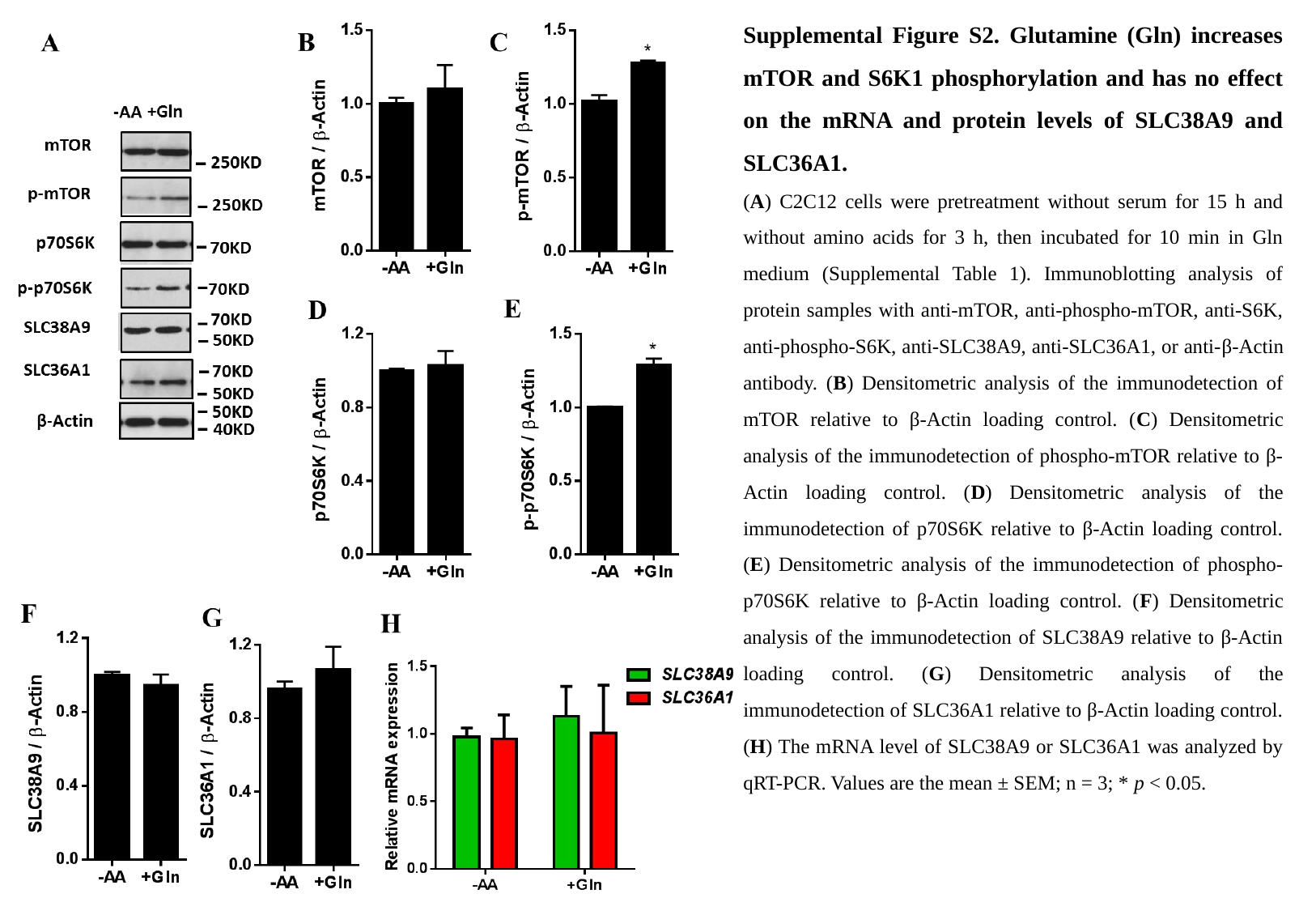

Supplemental Figure S2. Glutamine (Gln) increases mTOR and S6K1 phosphorylation and has no effect on the mRNA and protein levels of SLC38A9 and SLC36A1.
(A) C2C12 cells were pretreatment without serum for 15 h and without amino acids for 3 h, then incubated for 10 min in Gln medium (Supplemental Table 1). Immunoblotting analysis of protein samples with anti-mTOR, anti-phospho-mTOR, anti-S6K, anti-phospho-S6K, anti-SLC38A9, anti-SLC36A1, or anti-β-Actin antibody. (B) Densitometric analysis of the immunodetection of mTOR relative to β-Actin loading control. (C) Densitometric analysis of the immunodetection of phospho-mTOR relative to β-Actin loading control. (D) Densitometric analysis of the immunodetection of p70S6K relative to β-Actin loading control. (E) Densitometric analysis of the immunodetection of phospho- p70S6K relative to β-Actin loading control. (F) Densitometric analysis of the immunodetection of SLC38A9 relative to β-Actin loading control. (G) Densitometric analysis of the immunodetection of SLC36A1 relative to β-Actin loading control. (H) The mRNA level of SLC38A9 or SLC36A1 was analyzed by qRT-PCR. Values are the mean ± SEM; n = 3; * p < 0.05.

## Slide 3
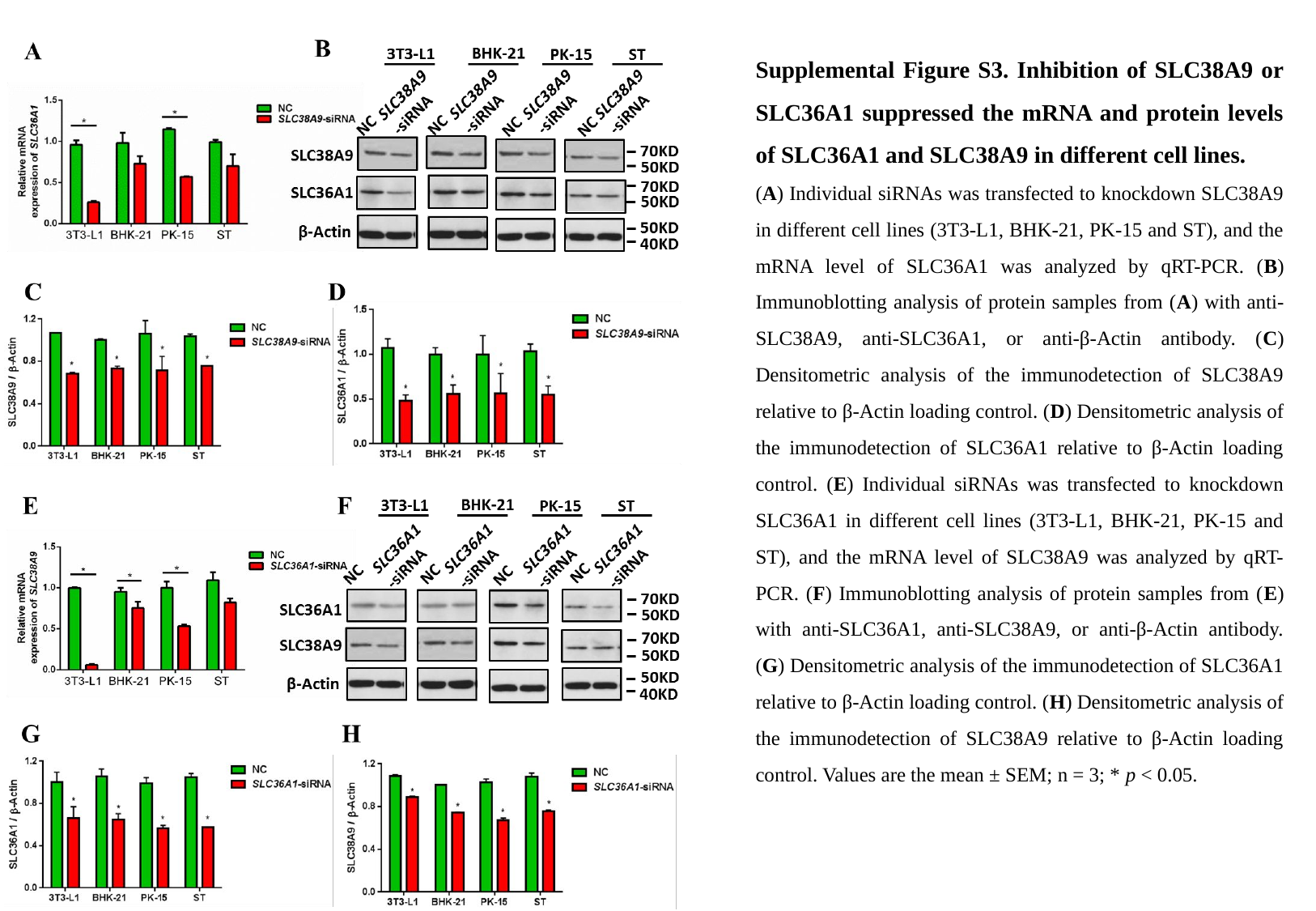

Supplemental Figure S3. Inhibition of SLC38A9 or SLC36A1 suppressed the mRNA and protein levels of SLC36A1 and SLC38A9 in different cell lines.
(A) Individual siRNAs was transfected to knockdown SLC38A9 in different cell lines (3T3-L1, BHK-21, PK-15 and ST), and the mRNA level of SLC36A1 was analyzed by qRT-PCR. (B) Immunoblotting analysis of protein samples from (A) with anti-SLC38A9, anti-SLC36A1, or anti-β-Actin antibody. (C) Densitometric analysis of the immunodetection of SLC38A9 relative to β-Actin loading control. (D) Densitometric analysis of the immunodetection of SLC36A1 relative to β-Actin loading control. (E) Individual siRNAs was transfected to knockdown SLC36A1 in different cell lines (3T3-L1, BHK-21, PK-15 and ST), and the mRNA level of SLC38A9 was analyzed by qRT-PCR. (F) Immunoblotting analysis of protein samples from (E) with anti-SLC36A1, anti-SLC38A9, or anti-β-Actin antibody. (G) Densitometric analysis of the immunodetection of SLC36A1 relative to β-Actin loading control. (H) Densitometric analysis of the immunodetection of SLC38A9 relative to β-Actin loading control. Values are the mean ± SEM; n = 3; * p < 0.05.

## Slide 4
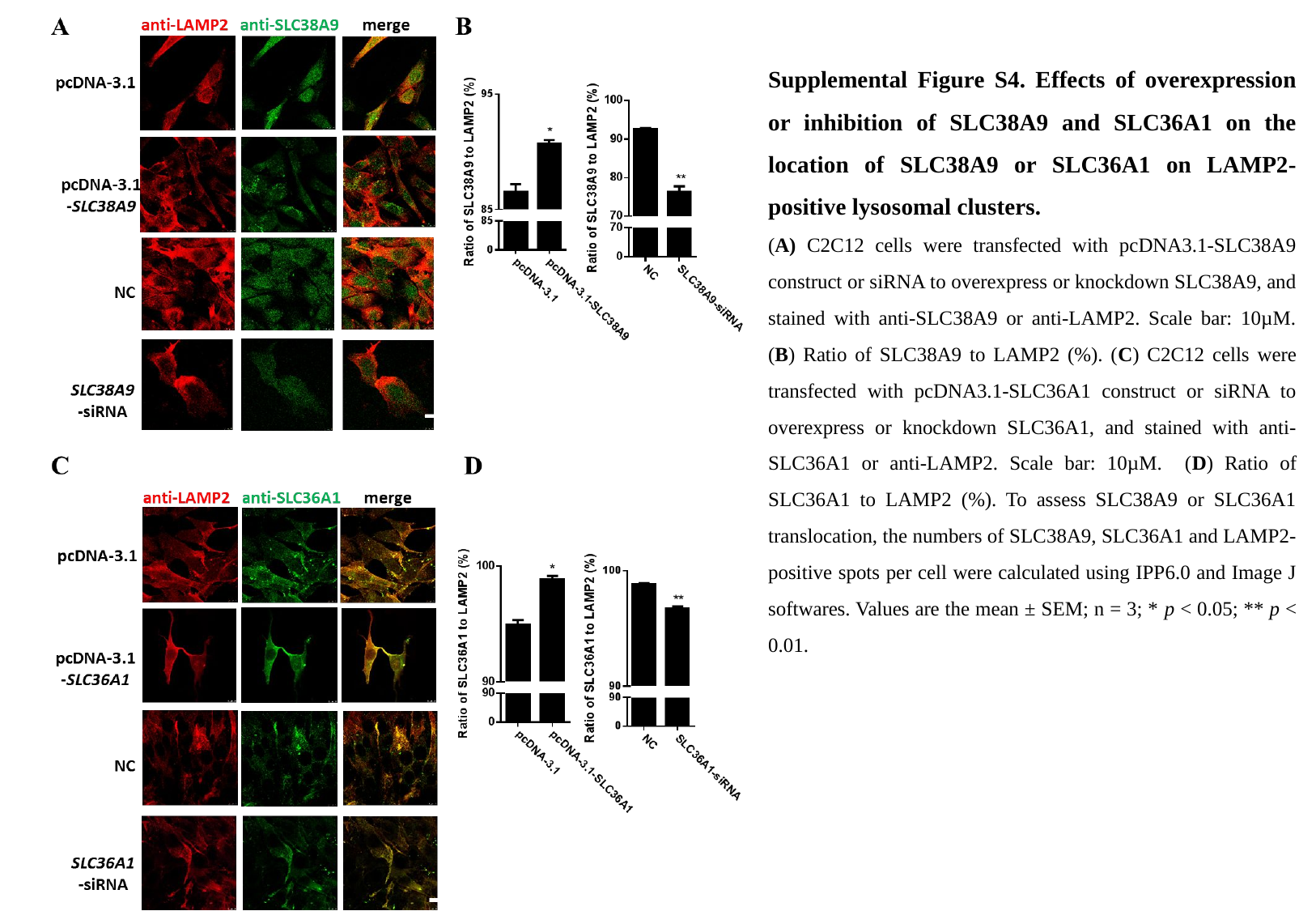

Supplemental Figure S4. Effects of overexpression or inhibition of SLC38A9 and SLC36A1 on the location of SLC38A9 or SLC36A1 on LAMP2-positive lysosomal clusters.
(A) C2C12 cells were transfected with pcDNA3.1-SLC38A9 construct or siRNA to overexpress or knockdown SLC38A9, and stained with anti-SLC38A9 or anti-LAMP2. Scale bar: 10µM. (B) Ratio of SLC38A9 to LAMP2 (%). (C) C2C12 cells were transfected with pcDNA3.1-SLC36A1 construct or siRNA to overexpress or knockdown SLC36A1, and stained with anti-SLC36A1 or anti-LAMP2. Scale bar: 10µM. (D) Ratio of SLC36A1 to LAMP2 (%). To assess SLC38A9 or SLC36A1 translocation, the numbers of SLC38A9, SLC36A1 and LAMP2-positive spots per cell were calculated using IPP6.0 and Image J softwares. Values are the mean ± SEM; n = 3; * p < 0.05; ** p < 0.01.

## Slide 5
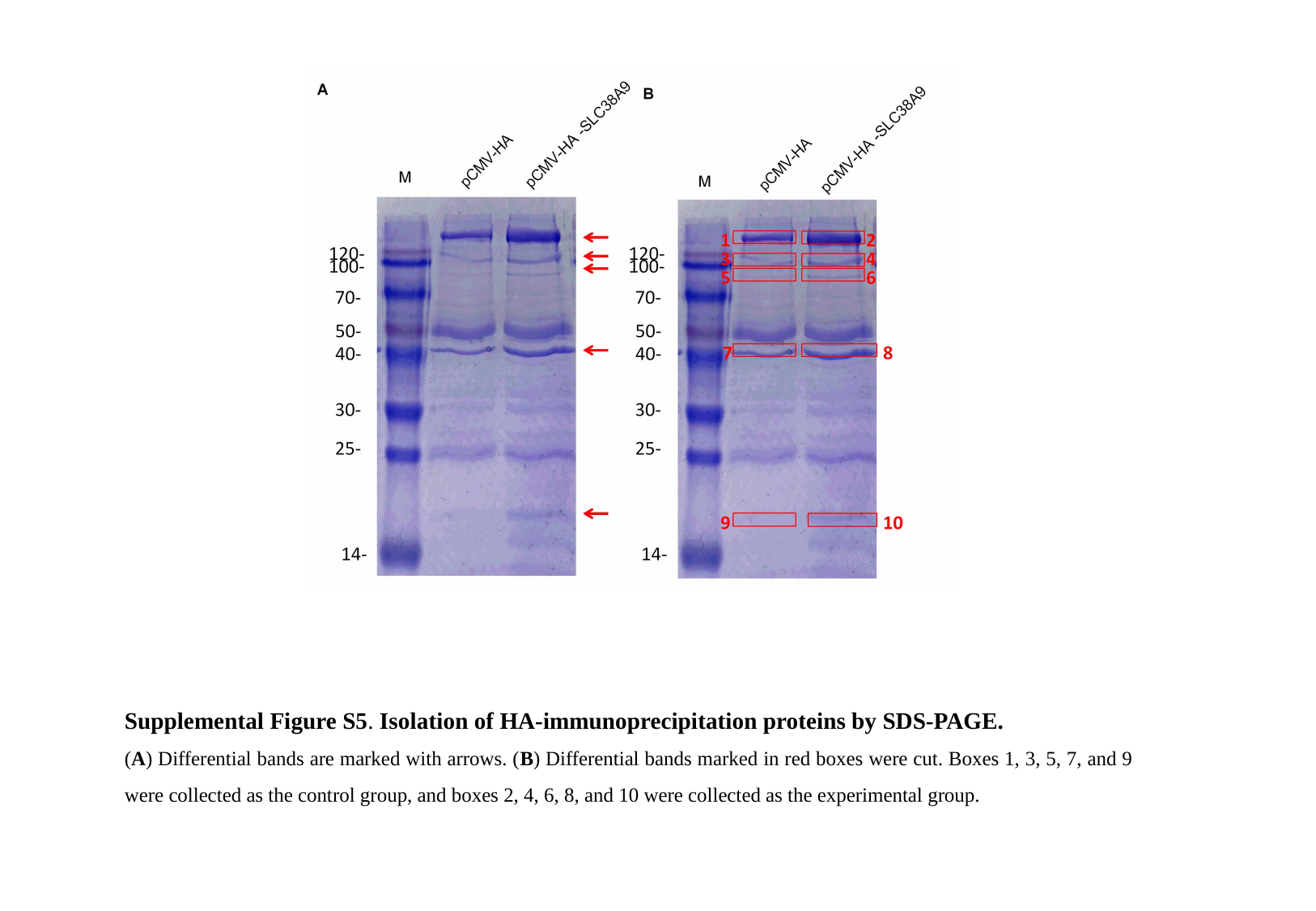

Supplemental Figure S5. Isolation of HA-immunoprecipitation proteins by SDS-PAGE.
(A) Differential bands are marked with arrows. (B) Differential bands marked in red boxes were cut. Boxes 1, 3, 5, 7, and 9 were collected as the control group, and boxes 2, 4, 6, 8, and 10 were collected as the experimental group.

## Slide 6
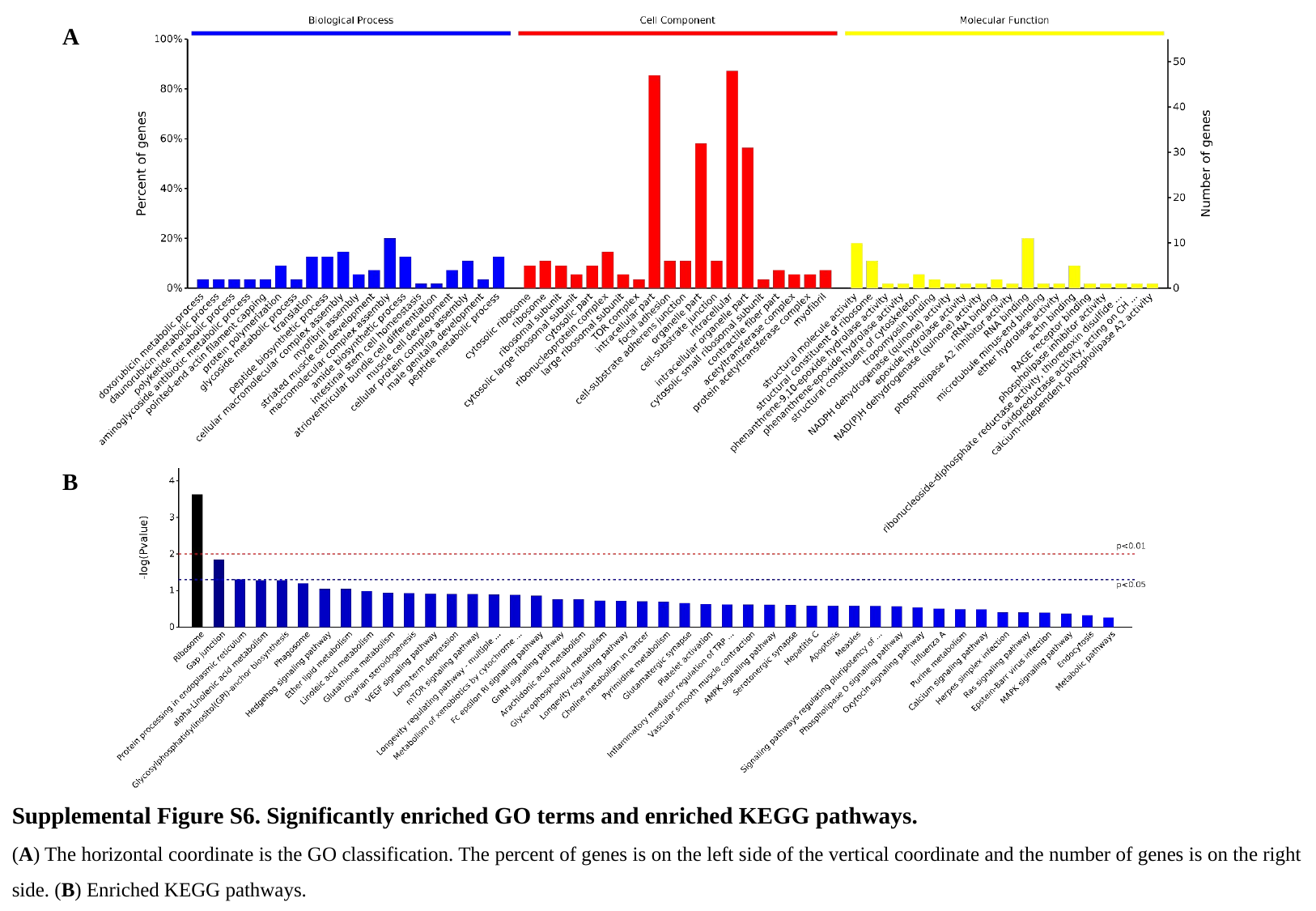

A
B
Supplemental Figure S6. Significantly enriched GO terms and enriched KEGG pathways.
(A) The horizontal coordinate is the GO classification. The percent of genes is on the left side of the vertical coordinate and the number of genes is on the right side. (B) Enriched KEGG pathways.
